# Tocilizumab: The Key to Stop Coronavirus Disease 2019 (COVID-19)-Induced Cytokine Release Syndrome (CRS)?

**DOI:** 10.3389/fmed.2020.571597

**Published:** 2020-10-26

**Authors:** Danfei Liu, Tongyue Zhang, Yijun Wang, Limin Xia

**Affiliations:** Hubei Key Laboratory of Hepato-Pancreato-Biliary Diseases, Department of Gastroenterology, Institute of Liver and Gastrointestinal Diseases, Tongji Hospital of Tongji Medical College, Huazhong University of Science and Technology, Wuhan, China

**Keywords:** COVID-19, cytokine release syndrome, interleukin-6, tocilizumab, SARS-CoV-2

## Abstract

The COVID-19 disease is an unprecedented international public health emergency and considerably impacts the global economy and health service system. While awaiting the development of an effective vaccine, searching for the therapy for severe or critical COVID-19 patients is essential for reducing the mortality and alleviating the tension of the health service system. Cytokine release syndrome (CRS) induced by elevated interleukin-6 was recognized to underscore the pathology of severe COVID-19 patients. Inhibiting CRS by agents suppressing IL-6 may relieve symptoms, shorten the hospital stay and reduce the need for oxygen therapy. Although evidence from randomized, double-blinded clinical trials is still lacking, the IL-6R inhibitor tocilizumab (TCZ) has shown some clinical benefits in the treatment of severe COVID-19 patients and have been included in clinical guidelines. In this review, we focused on the possible mechanisms of TCZ in the treatment of CRS and highlighted some significant considerations in the use of TCZ to treat COVID-19 patients.

## Introduction

The novel zoonotic coronavirus disease, caused by severe acute respiratory syndrome-coronavirus 2 (SARS-Cov-2), has rapidly transmitted across the continents in the past several months and eventually evolved into a pandemic, which infected over 5,000,000 patients worldwide and resulted in over 300,000 deaths (WHO). SARS-CoV-2 gains entry into host cells through interaction with angiotensin-converting enzyme-2 (ACE2). ACE2 is ubiquitously expressed in numerous tissues; however, lung alveolar epithelial cells are considered the primary targets. Subsequently, the virus rapidly replicates in the host, causing massive epithelial and endothelial cell apoptosis and vascular leakage, triggering the release of exuberant pro-inflammatory cytokines and chemokines. The excessive immune reaction produced by SARS-CoV-2 infection in the host can lead to the “cytokine release syndrome” (CRS). CRS can induce an extensive tissue damage and be life-threatening in severe COVID-19 (1, 2).

While the majority of the patients infected by SARS-Cov-2 only developed a mild to modest symptoms and may recover in several days, up to 20% patients demonstrated severe pneumonia and fever, which develop into acute respiratory distress syndrome (ARDS) and need admission into the intensive care unit (3). As the surge in the number of critical patients overwhelmed the medical service system, an effective strategy to reduce the mortality and shorten the average length of stay in ICU will significantly improve the treatment of COVID-19.

Compared with moderately ill cases, severe cases more frequently developed lymphopenia, and hypoalbuminemia, with higher levels of alanine aminotransferase, lactate dehydrogenase, C-reactive protein, ferritin, and D-dimer as well as markedly higher levels of cytokines including IL-2R, IL-6, IL-10, and TNF-α (4). The indications above are characteristic of CRS, which is common in critical patients and underlies the pathology of ARDS (5).

A meta-analysis including nine studies found that patients with severe COVID-19 had a significantly higher serum IL-6 levels compared to non-severe patients, and increased IL-6 levels are correlated with elevated mortality in patients (6). Elevated IL-6 levels might also serve as a predictive biomarker for disease severity. The optimum critical point of IL-6 was determined as 24.3 pg/ml in severe COVID-19 group. When IL-6 was over 24.3 pg/ml, the severity of COVID-19 could be predicted with sensitivity and the speciality of 73.3 and 89.3%, respectively. And when combined IL-6 with D-dimer, the sensitivity reached to 93.3% by parallel testing, the speciality was increased to 96.4% by tandem testing (7). IL-6 showed a significant kinetics changes in COVID-19 cases with fatal outcome. On admission, in severe or critical patients, significantly higher levels of IL-6 were found in non-survivors (severe: 9.7 pg/mL, critical: 10.5 pg/mL) than in survivors (severe: 7.2 pg/mL, critical: 6.2 pg/mL). Survivors had comparable levels of IL-6 during hospitalization, whereas IL-6 showed an upward trend with varying degrees in non-survivors at end-hospitalization. A 1.2-, 1.5-, and 2.2-fold increase was found in mild/moderate, severe, or critical cases compared with on admission, respectively (8). These studies indicated the central role of IL-6 in the CRS and the exacerbation of COVID-19 patients, highlighting the possibility of targeting the IL-6 pathway in the treatment of patients with COVID-19.

Therefore, an effective way to modulate the immune overactivation and to suppress the CRS may reduce the mortality of severely ill patients. Tocilizumab (TCZ) is a humanized monoclonal antibody targeting the IL-6 receptor and is approved by FDA for the management of CRS invoked by CAR-T therapy. TCZ showed promising efficacy in severe CRS demonstrated by a response as defined achieved by 69% patients within 14 days after one or two doses of TCZ. Moreover, TCZ is safe for both pediatric and adult patients, as no adverse reactions observed in patients with CAR T cell-induced CRS (9). The involvement of CRS in the COVID-19 progression is evidenced by the increased pro-inflammatory factors [IL-6, IL-1, IL-2, IL-7, IL-10, granulocyte-colony stimulating factor, interferon-γ-inducible protein 10, monocyte chemoattractant protein 1, macrophage inflammatory protein-1 alpha, and TNF-α] observed in severe COVID-19 patients (10–12). In addition, COVID-19 can progress in the same fashion as the respiratory signs and symptoms associated with CRS (13). TCZ has therefore been hypothesized to improve COVID-19 patients' condition through blocking exuberant and dysfunctional systematic inflammation. Although its efficacy in treating severely ill patients has not confirmed, the guidelines issued by China and Italy have already recommended TCZ for the treatment of critical COVID patients with elevated IL-6. Given the efficacy and safety of TCZ in CRS and the pivotal role of CRS in COVID-19, this review focused mainly on the existing evidence concerning the mechanisms of TCZ, and to clarify the considerations in clinical application.

## The Mechanisms of TCZ

CRS referred to an activation cascade of auto-amplifying cytokine production due to dysfunctional immune response involving the continual activation and expansion of lymphocytes and macrophages (14). CRS was regarded as the prominent cause of fatality in the previous SARS-CoV and MERS-CoV infections (15). Similarly, a cytokine profile resembling CRS in critical patients with COVID-19 was also documented (4, 10). Furthermore, the biopsy of the patients died from the COVID-19 showed the bilateral diffuse alveolar damage with cellular fibromyxoid exudates and interstitial mononuclear inflammatory infiltrates dominated by lymphocytes, suggesting the role of overactivated inflammatory cells in the pathology of patients with COVID-19 (13). A recent retrospective, multicenter study of 150 confirmed COVID-19 cases revealed that elevated IL-6 was a predictor of mortality, indicating that IL-6 played a central role in the hyperinflammation and CRS in the critical patients (11).

Interleukin-6 (IL-6) is a pleiotropic cytokine that exerted multiple roles in the immune system (16). It can be produced by almost all stromal cells and immune system cells, such as B lymphocytes, T lymphocytes, macrophages, monocytes, dendritic cells, mast cells and other non-lymphocytes, such as fibroblasts, endothelial cells (17). But the primary source of interleukin-6 comes from the monocytes and macrophages at the sites of inflammation (18, 19). The expression of IL-6 was mainly induced by interleukin-1α, tumor necrosis factor (TNF)-α and other stimuli including virus infections, bacterial products and factors secreted by necrotic cells (19). The receptor of IL-6 consisted of two kinds of receptor, the membrane-bound receptor (mIL-6R) and soluble receptor (sIL-6R). As the receptor of IL-6 is an incompetent receptor in signaling transduction, the complex formed by IL-6 and IL-6R need a second receptor, gp130, to initiate transmembrane signaling (20, 21). Notably, while membrane-bound IL-6R can only be detected on hepatocytes and certain immune cells, the gp130 is ubiquitously expressed on the surface of all cells (22, 23). By binding to different forms of IL-6R, IL-6 signals through two distinct pathways, referred to as the classical signaling and the trans-signaling (24). In classical signaling, IL-6 binds to the membrane-bound IL-6R and then recruited the gp130, whose dimerization activated the downstream signaling including the STAT1, STAT3, PI3K, and MAPK pathways. In trans-signaling, IL-6 binds to the soluble IL-6R (sIL-6R) and forms a soluble complex, interacting with a dimer of gp130 and enabling cells without the expression of IL-6R to respond to IL-6 signaling, which widens the cell type affected by IL-6 signaling (16).

Under homeostatic conditions, the serum IL-6 concentrations in healthy individuals are in the range of 1–5 pg/ml (18). Interestingly, the serum concentrations of soluble gp130 and sIL-6R are in the range of 40–70 ng/ml and 400 ng/ml, respectively (25). Once secreted, IL-6 was neutralized by binding to sIL-6R, which has a higher affinity to IL-6, and then associating with sgp130. Therefore, the serum sIL-6R and sgp130 may serve as a buffer against elevated IL-6 concentration. In physiological context, IL-6 functions in a paracrine manner due to the existence of the IL-6 buffer (24). Under inflammatory conditions, the concentration of IL-6 surged, surpassing the buffer capacity and then activating the trans-signaling, which lead to hyperinflammation status and eventually result in CRS.

**Figure 1:**
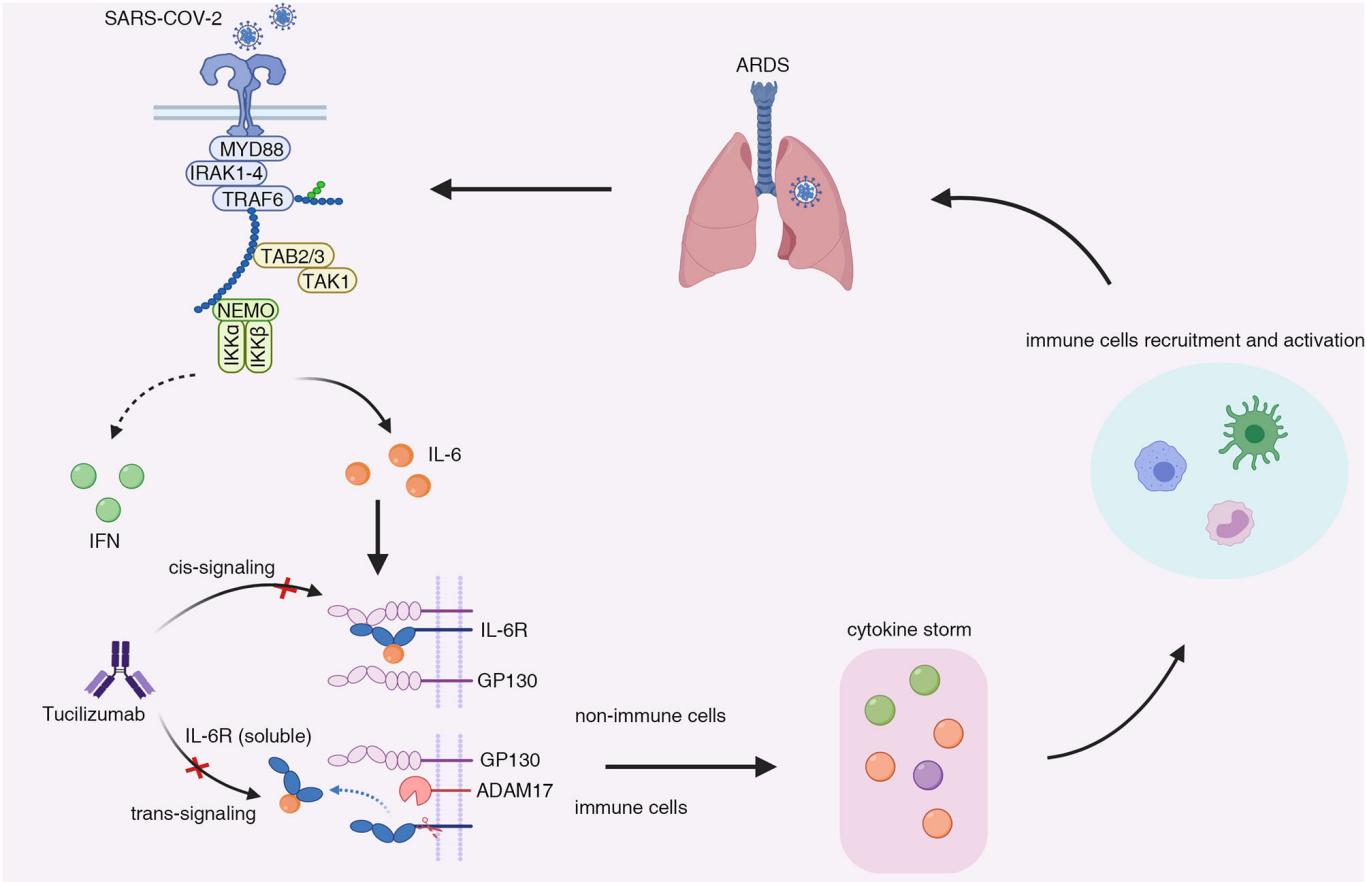
The possible mechanisms underlying the cytokine release syndrome induced by IL-6 elevation in COVID-19 patients. In severe patients infected with SARS-Cov-2, antigen-presenting cells (e.g., dendritic cells and monocytes) demonstrated low interferon response while maintaining pro-inflammatory cytokines production. Besides, the count of CD4+ T cells and CD8+ T cells also decreased in severe COVID-19 cases. The impaired anti-viral responses resulted in delayed viral clearance and massive viral replication, leading to persistent production of pro-inflammatory cytokine (e.g., IL-1β, IL-6, TNF-α). A large amount of IL-6 was secreted by non-immune cells upon stimulation of IL-6 through trans-signaling, which formed a positive feedback or so-called “inflammation amplifier.” The “amplifier” resulted in the surge in pro-inflammatory cytokines and chemokines (IL-6, IL-1β, IP-10, IL-2, IL-10, IFNγ, MCP1, GM-CSF, TNF.), contributing to the development of cytokine release syndrome and the recruitment of inflammatory cells, including monocytes, neutrophils, and macrophage cells. The infiltrates of inflammatory cells in lung led to acute respiratory dysfunction syndrome and the exacerbation of the COVID-19. MYD88, myeloid differentiation primary response 88; IRAK1-4, IL-1R-associated kinase family kinase 1-4; TRAF6, tumor necrosis factor receptor-associated factor 6; TAB 2/3, TGF-β-activated kinase 1-binding protein 2/3; TAK1, TGF-β-activated kinase 1; NEMO, NF-κB essential modulator; IFN, interferon; IL-6, Interleukin-6; GP130, glycoprotein 130; IL-6R, Interleukin-6 receptor; ADAM17, ADAM metallopeptidase domain 17; ARDS, acute respiratory distress syndrome.

Although the exact mechanisms remained unknown, the so-called “IL-6 amplifier” may explain the CRS in severe COVID-19 patients. In COVID-19 patients, innate immune cells, including dendritic cells and macrophages, were activated upon sensing of coronaviruses *via* toll-like receptors, which induced the expression of pro-inflammatory cytokines (e.g., IL-1, IL-6, TNF-a) through engagement with NF-κB signaling (26, 27). Besides, various cell types infected with SARS-CoV-2 demonstrated very low IFN-I or IFN-III and limited ISG response, while maintaining high chemokine and pro-inflammatory cytokine production marked by IL-6, IL-1RA (28). Furthermore, the count of CD4+ T cells, CD8+ T cells, and B cells negatively correlated with the severity of illness (29, 30). Serum IL-6, IL-10, and TNF-α concentration were inversely correlated with T cell numbers (31), and high levels of lymphocyte apoptosis in the spleens and lymph nodes was found in patients died from COVID-19 (32). Whether IL-6 plays a role in the decrease of T cells needs further investigation. The impaired interferon response and the reduction of T cells lead to delayed viral clearance and massive viral replication, promoting the synthesis and secretion of IL-6 in innate immune cells. The non-immune cells responded to elevated IL-6 through IL-6 trans-signaling, and expressed high concentrations of chemokines, growth factors, and IL-6, which forms a positive feedback loop or “inflammation amplifier” (27). The “amplifier” may result in consistent elevation of pro-inflammatory chemokines and cytokines and persistent monocyte infiltrates, causing CRS and acute respiratory dysfunction syndrome (Figure 1). IL-6 can act on a large number of cells and tissues to get involved with the progression of COVID-19. IL-6 contributed to increased angiogenesis activity and vascular permeability by induction of vascular endothelial growth factor (VEGF) (23), which further exacerbate ARDS in severe patients. IL-6 is essential for B cells to proliferation, differentiation and antibodies production. IL-6 is especially needed when B cells are activated by antigen and differentiate into IgM, IgG and IgA antibodies (33), which may be associated with the seroconversion during COVID-19 pathogenesis (34). IL-6 induces cytotoxic T lymphocyte (CTL) activity, promotes the lineage and function of Th17 cell and the development of self-reactive pro-inflammatory CD4 T cell response, whereas inhibits the induction of regulatory T cell (Treg) (35), which may explain the hyperactivated pro-inflammatory T cells (13) and decreased regulatory T cells (36) observed in critically ill patients. IL-6 also induces hepatocytes to synthesize acute phase reactive protein, especially Serum amyloid A (SAA) and C-reactive protein (CRP) (37), which were significantly elevated in COVID-19 patients (37). IL-6 trans-signaling mediates inflammation leading to cardiovascular diseases (38) that is a complication in COVID-19 patients (10). Thus, targeting the IL-6 signaling pathway may reverse the hyperinflammation status and curb the CRS, potentially be the effective and safe way to reduce mortality of COVID-19.

TCZ was the first monoclonal antibody developed to block the IL-6 signaling pathways, which functions by directly binding to the mIL-6R and sIL-6R to prevent IL-6 from interacting with IL-6R (39). The safety and effectiveness of TCZ in blocking IL-6 have been proved in therapy for rheumatic disease and CAR-T induced CRS (39). CAR-T cells are T cells extracted from the patients with cancer and modified to enhance its recognition and eradication of tumor cells (40). However, around 70% of patients receiving CD19 CAR-T therapy develop CRS, with symptoms including fever, dyspnea, and hypotension and abnormities in laboratory parameters, such as cytopenia, hypofibrinogenemia and elevated cytokines. As an elevation of IL6 accompanies CRS, TCZ was used in the management of CAR-T induced CRS and was eventually approved by the FDA for its efficacy. As critical patients with COVID-19 and patients developing CRS after CAR-T therapy share similarities in clinical presentation and laboratory findings, it is reasonable to speculate that TCZ may also be effective in the treatment of patients with COVID-19.

## The TCZ in the Treatment of COVID-19

The efficacy of TCZ in the treatment of critically ill patients with COVID-19 has aroused great interest given the success of TCZ in the management of CAR-T therapy-induced CRS. Although the data from randomized, double-blind, placebo-controlled clinical trials are still lacking, several retrospective studies around the world have reported promising results, which was summarized in Table 1. While the reported mortality for critical patients was ~60.5% (50), a clinical trial in China including 21 patients (17 severe/4 critical) treated by TCZ showed favorable results that 15 out of 20 patients (75.0%) had improved respiratory condition and all patients have been discharged on average 15.1 d after TCZ treatment. The primary abnormality of 21 severe patients on the initial chest CT was plaque-like, ground glass opacities and focal consolidation. The ground glass opacities increased in size, extent, and severity within the first 7 d after admission before receiving TCZ. But the lesions were clearly absorbed in 19 patients (90.5%) after the treatment with TCZ. Among them, 13 (61.9%) patients were discharged within 2 wk after TCZ, and six were discharged within 3 wk (43). It indicated that TCZ may exert anti-fibrotic effect in severe COVID-19 patients.

**Table 1:** The clinical trials concerning the use of TCZ in the treatment of severe COVID-19 patients.

	**Study type**	**Patients (*n*)**	**Median age (age range)**	**Comorbidities**	**Dosage and administration route**	**Criteria for TCZ administration**	**Outcomes**
Rand Alattar (41)	retrospective study	25 (23M, 2F) all critically ill	58y, 50–63 (IQR)	Diabetes Mellitus (48%) CKD (16%) Malignancy (4%) CVD (12%)	median dose: 1 median total dose of 5.7 mg/kg 9 received two or more doses i.v.	↑ CRP and require supportive care in ICU	9(36%) discharged; 3(12%) died; 13(52%) in ICU; Patients on invasive ventilation declined from 21 (84%) at day 0 to 7 (28%) on day 14 (*P* = 0.001)
Pan Luo (42)	Retrospective study	15 (12M, 3F) 2 moderately ill 6 seriously ill 7 critically ill	73y, 62–80	CVD (66.7%) Diabetes Mellitus (26.7%)	5 received two or more doses The dose of TCZ was the range from 80 to 600 mg per time.	↑ CRP and ↑ IL-6	**Critically ill patients:** 3/7(42.9%) died, 1/7 (14.3%) aggravated, all of which received only one dose **Seriously ill patients:** 1/6(16.7%)
Xiaoling Xu (43)	Retrospective study	21 (18M, 3F) 17 severe 4 critical	56.8y, 25–88	Hypertension (42.9%) Diabetes (23.8%) CHD (9.5%) Auricular fibrillation (4.8%) Bronchiectasis (4.8%) Brain infarction (4.8%) COPD (4.8%) CKD (4.8%)	4–8 mg/kg body weight 18 patients (85.7%) received one dose 3 patients (14.3%) had a second administration at the same dose due to fever within 12 h i.v.	a) persistent fever b) the condition changes from mild to severe (including high- risk factor for severe cases) c) diffuse lung opacities on CT scans d) ↑ IL-6	15/20 patients (75.0%) had lowered their oxygen intake, and 1 patient needed no oxygen therapy; All patients have been discharged on average 15.1 d after giving TCZ
S. Sciascia (44)	Prospective open, single-arm multicenter study	63 (56M, 7F)	62.6 ± 12.5	Hypertension (38.0%) Diabetes mellitus (9.5%) COPD (4.7%) Heart disease (7.0%)	52 received a second administration within 24 h i.v. (8 mg/ kg) or s.c. (324 mg)	a) confirmed COVID-19 b) SaO2 <93%, or Pao2/Fio2 <300 mm Hg; c) pro-inflammatory and pro-thrombotic profile (at least 3 of the following): CRP > × 10 normal values; ferritin >1,000 ng/ml; D-dimer × 10 normal values; LDH × 2 the upper limits	The overall mortality was 11% No differences between the route of administration were reported in terms of mortality TCZ administration within 6 days from admission in the hospital was associated with an increased likelihood of survival
Ruggero Capra (45)	Retrospective study, single center	85 (64M, 21F) control:23 (19M, 4F) TCZ:62 (45M, 17F)	65y, 54.5–73(IQR) TCZ group: 63y, 54–73(IQR) Control group: 70y, 55–80(IQR)	TCZ Group: Hypertension (46%) Diabetes (14%) Hearth disease (14%) Control group: Hypertension (48%) Diabetes (22%) Hearth disease (26%)	33(53%) 400 mg i.v. 2(3.5%) 800 mg i.v. 27(43.5%) 324 mg s.c.	a) Consecutive patients with respiratory failure and without mechanical ventilation b) respiratory rate ≥30 breaths/min; c) peripheral capillary SaO2 ≤ 93% or PaO2/FiO2 < =300 mmHg	Death:TCZ: 2/62; Control: 11/23; Patients receiving TCZ showed significantly greater survival rate as compared to control patients
Paola Toniati (46)	Retrospective study	100 (88 M, 12 F) 43 (ICU) 57 (general ward) NIV	62y, 57–71(IQR)	Hypertention (46%) Obesity (31%) Diabetes (17%) CVD (16%)	8 mg/kg by two consecutive intravenous infusions 12 h apart A third infusion was optional based on clinical response	a)rapidly progressive respiratory failure b) refractory to pharmacological therapy c) ventilatory support	In 57 patients, 37 (65%) improved and suspended non-invasive ventilation (NIV), 7 (12%) patients remained stable in NIV, and 13 (23%) patients worsened, of which 10 (17.5%) died, 3 were admitted to ICU. In 43 patients in ICU, 32 (74%) improved (17 taken off the ventilator and discharged to the ward), 1 (2%) remained stable and 10 (24%) died 77 (77%) patients (61 showed a significant improvement on chest x-ray and 15 discharged). 23 (23%) patients worsened, of whom 20 (20%) died.
Marta Colaneri (47)	Prospective open, single-arm multicenter study	SOC group: 91 (63M, 28F) TCZ: 21 (19M, 2F) only the 42 patients (TCZ:21, SOC:21) matched by propensity score matching were included for analysis.	SOC group: 63.74 ± 16.32 TCZ group: 62.33 ± 18.68	NA	The first administration was 8 mg/kg (up to a maximum 8 mg/kg, up to 800 mg per dose), repeated after 12 h if no side effects were reported	a) CRP > 5 mg/dl; b) PCTI <0.5 ng/mL; c) PaO2/FiO2 < =300 mmHg <300; d) ALT <500 U/L.	Treatment with TCZ did not significantly affect ICU admission and 7-days mortality rate
Mathilde Roumier (48)	Retrospective study	TCZ group: 30 (24M, 6F) Control group: matched for age, gender and disease severity using the inverse probability of treatment weighted methodology	NA	NA	8 mg/kg; renewable in case of insufficient response to therapy; i.v.	a) <80y; b) >5 days of prior disease duration; c) severe (i.e., requiring strictly over 6 L/min of oxygen therapy); d) rapidly deteriorating pnuemonia (i.e., increase by more than 3 L/min of oxygen flow within the previous 12 h); e) high CRP levels	TCZ significantly reduced the requirement of subsequent mechanical ventilation In patients treated outside the ICU, TCZ significantly reduced the risk of subsequent ICU admission In TCZ group, 3 (10%) had died, 4/7 (57%) discharged from the ICU and 6/30 (20%) from hospital
T. Klopfenstein (49)	Retrospective case-control study	45 TCZ group:20 ST group:25	TCZ group: 76.8 (52–93)	Hypertension (55%) CVD (70%) Diabetes mellitus (25%) Malignancy (35%) COPD (20%)	NA	a) failure of standard treatment; b) time to symptom onset >7 days; c) oxygen therapy ≥ 5 liters/min; d) > 25% of lung damages on chest CT scan; e) ≥ 2 parameters of inflammation or biological markers of mortality (with a high level)	TCZ may decrease the need for invasive mechanical ventilation, reduce the number of ICU admissions and/or mortality in patients with severe SARS-CoV-2 pneumonia
			ST Group: 70.7 (33–96)	Hypertension (44%) CVD (68%) Diabetes mellitus (32%) COPD (4%) Malignancy (8%)			

*↑elevated; ↓decreased; TCZ, TCZ; SaO2, Oxygen saturation; CRP, C-reactive protein; CVD, cardiovascular diseases; ALT, alanine aminotransferase; PCTI, Procalcitoni*.

Another clinical trial in Italy included 100 consecutive patients with confirmed COVID-19 pneumonia and ARDS requiring ventilatory support, all of which were given TCZ intravenously in addition to routine therapy (46). Before TCZ administration, all patients manifested lymphopenia and high levels of inflammatory markers, including C-reactive protein (CRP), fibrinogen, ferritin and interleukin 6 (IL-6). At 24–72 h after TCZ administration, 58 patients (58%) showed a rapid improvement of clinical and respiratory condition, 37 (37%) stabilized and 5 (5%) worsened (of whom four died). Ten days after TCZ treatment, the laboratory results returned to the normal range, and the respiratory condition was improved or stabilized in 77 (77%) patients, of whom 61 showed a significant radiological improvement and 15 were discharged from the hospital. Respiratory condition worsened in 23 (23%) patients, of whom 20 (20%) died. Similar results were yielded in a pilot prospective open, single-arm multicenter study involving 63 severe COVID-19 patients, who were included by pre-established inclusion criteria and whose clinical and laboratory parameters were prospectively collected (44). Consistently, the clinical symptoms and abnormal inflammation markers were quickly curbed after a short time of TCZ treatment. At day 14 after TCZ treatment, the overall mortality was 11% in this cohort, with only two patients still requiring mechanical ventilation compared to five at admission.

However, another prospective open, single-arm multicenter study reported negative results that TCZ administration reduced neither ICU admission nor mortality rate by comparing a cohort of 21 patients receiving TCZ to propensity-scores-matched 21 patients treated with Standard Of Care (SOC) (47). The differences in the current studies concerning the effectiveness of TCZ in the treatment of severe or critical COVID patients highlighted the urgent need for the evidence from a randomized clinical trial.

Several larger-scale prospective randomized-controlled clinical trials to assess the benefits of inflammatory cytokines inhibition by targeting IL-6 alone or in combination with other agents are still underway. Italian Pharmaceutical Agency (AIFA) approved a Phase II trial in 330 patients with COVID-19 induced ARDS using TCZ started on March 18, 2020. In the US, a total of four Phase 3, randomized, double-blind, placebo-controlled studies assessing efficacy and safety of TCZ (NCT04412772, NCT04356937, NCT04372186, NCT04320615, NCT04409262) were initiated. These clinical trials may further clarify the role of TCZ in the management of severe COVID-19 patients.

Notably, the optimal timing for the beginning of TCZ administration and the patients most likely to respond to TCZ remained to be resolved. As a previous study reported that IL-6 can either suppress or promote the viral replication (51), premature TCZ treatment may lead to the delay of viral clearance while delayed administration may undermine the effectiveness of anti-IL-6 therapy. The guideline issued in China recommended the use of TCZ in patients with extensive lung lesions, severe diseases and high IL-6 levels. Additionally, the prospective single-arm multicenter study reported that early TCZ initiation in patients with elevated inflammatory parameters is associated with a two-fold increased survival (HR 2.2, 95%CI 1.3–6.7, *p* < 0.05) (44). These abnormal inflammatory parameters are as followed: CRP > × 10 normal values; ferritin >1 000 ng/ml; D-dimer × 10 normal values; LDH × 2 the upper limits (44). Similarly, a recent study based on machine learning reported that LDH, lymphopenia and high-sensitivity C-reactive protein (hs-CRP) could predict the mortality of individual patients in advance with more than 90% accuracy (52). These studies may suggest that the administration of TCZ should be initiated as soon as possible when a hyperinflammatory status was monitored, and the critical patients with abnormal inflammatory indications such as elevated CRP, LDH and reduced lymphocytes are most likely to benefit from anti-IL-6R therapy.

The dosage and the administration route of TCZ in the management of patients with COVID-19 seemed to be equivocal. While currently no clinical trials have evaluated the correlation between the dosage of TCZ and the outcome of the patients, the strategy of TCZ was basically followed the guidelines of FDA for the treatment of CAR-T induced CRS. In several trials, most patients received only a single dose of TCZ, and two or more doses of TCZ were only given when needed (42, 43). However, in other trials, all patients received a second dose of TCZ as long as no side effects were observed (46, 47). The repeated doses (even repeated with a lower dose) of TCZ is more effective than a single dose at least in critically ill patients. Patients treated with repeated doses were clinical stabilization, while those treated with single dose were disease aggravation or death. Moreover, repetitive TCZ therapy may be beneficial for moderately ill patient with almost 90 times elevated IL-6 (42). Although in most of the trials the TCZ was given by intravenous infusion, subcutaneous injection of TCZ was also applied in some studies (44, 45). While subcutaneous administration of TCZ is not recommended for the treatment of CRS, a study observed no differences between the route of administration in terms of mortality (44). Considering the surge in the number of patients infected with SARS-Cov-2, the cost of antibody therapy and the drug availability, future studies should focus on the optimal dosage and administration route of TCZ. Currently, a 50-person clinical trial evaluating whether lower doses (80 or 200 mg) of TCZ would be effective in the treatment of COVID-19 pneumonitis in hospitalized, non-critically ill patient is ongoing (NCT04331795). According to the Chinese guideline, the first dose of TCZ was 4–8 mg/kg body weight, and the recommended dose was 400 mg through an i.v. drip up to a maximum of 800 mg. For patients with poor efficacy of the first dose, an additional dose (the dose is the same as before) can be applied after 12 h, with a maximum of two cumulative doses and a maximum of 800 mg for a single dose.

Given the role of IL-6 signaling pathway in the immunity, the safety of the anti-IL-6R therapy is also a major consideration. For the lacking of clinical data concerning the safety of TCZ in the treatment of COVID-19, the safety profile was mainly obtained from previous studies in the treatment of rheumatic diseases with TCZ. The adverse effects reported include serious infections, elevations in serum concentrations of transaminases, pancreatitis, gastrointestinal perforations, increased serum lipid concentrations (LDL and triglycerides), and the development of anti- drug antibodies (39). In the data from available clinical trials, most trials reported no severe-to-moderate adverse events directly related to TCZ infusions (Table 1). And the 100-person trials reported three cases of severe adverse events, with two developed septic shock and died finally and one gastrointestinal perforation (46). A 25-persons study reported 92% patients experienced at least one adverse event and TCZ treatment may be associated with opportunist infections, while no TCZ therapy was suspended due to adverse effects (41). Of note, in patients with a history of gastrointestinal perforation, intestinal ulcers or diverticulitis or patients with confirmed infections, TCZ should be used with caution (39, 53). In conclusion, the adverse effects of TCZ were generally mild and manageable if used under close monitoring and great caution.

## Discussion

CRS is well-recognized to underline the pathophysiology of severe COVID-19 patients. Interleukin-6 induced by SARS-Cov2 infection positively correlated with the severity of COVID-19 patients. Suppressing IL-6 signaling pathways by has also shown clinical benefits in some retrospective studies. However, the mechanisms between SARS-Cov-2 infection and CRS are largely unknown. The subsets of patients inclined to develop CRS after SARS-Cov-2 infection remained undefined. While aged patients and patients with comorbidities are susceptible to CRS invoked by SARS-Cov2 and more likely to develop severe cases due to relatively impaired immune response, some younger patients without pre-existing risk factors can also develop the severe disease (54). Besides, the role of IL-6 in CRS and how it interacts with the innate and adaptive immune system in the settings of COVID-19 still needs further investigation.

TCZ is a humanized monoclonal antibody IgG1 anti-human receptor for IL-6, obtained from Chinese hamster ovary (CHO) cells by recombinant DNA technology. TCZ is therapeutically applied in rheumatoid arthritis, but Chinese government have stated that it can be prescribed for COVID-19 patients with severe lung damage and high IL-6. TCZ has shown good efficacy in several clinical trials so far. TCZ also has inevitable limitations and clinical defects in the treatment of COVID-19. For example, Optimal COVID-19 infection management with TCZ is not achieved during hyperglycaemia in both diabetic and non-diabetic patients that TCZ administration neither decreases IL-6 levels nor attenuates risk of severe outcomes (55). Similarly, the use of TCZ fails to decrease the mortality rate at 30 days of patients with severe COVID-19 acute respiratory distress syndrome with hyperinflammation, after correction for pre-existing comorbidities and the need for respiratory support (56). In addition, adverse reactions of TCZ in other long-course treatments need to be noted. (1) Serious infections: including pneumonia, urinary tract infection, cellulitis, herpes zoster, gastroenteritis, diverticulitis, sepsis and bacterial arthritis. (2) Gastrointestinal perforations: frequently occurred when taking concomitant non-steroidal anti-inflammatory drugs (NSAIDs), corticosteroids, or methotrexate at the same time, including generalized purulent peritonitis, lower GI perforation, fistula and abscess. (3) Infusion reactions: hypertension, headache and skin reactions. (4) Anaphylaxis: occurred during the second to fourth infusion of TCZ. (5) Abnormal laboratory indexes: including thrombocytopenia, elevated liver enzymes and elevated lipid parameters (total cholesterol, LDL, triglycerides) (53). It needs to be further demonstrated that whether the adverse reactions will occur during the short-course treatment of COVID-19. Medication guide from China and the US FDA reveals that TCZ actively crosses the placenta in late pregnancy and may affect the fetal immune response as well as patients aged ≥ 65 years treated with TCZ have a higher rate of severe infection than patients ≤ 65 years. Care therefore should be taken in the treatment of pregnant woman, lactation women and older adults.

Although TCZ has been recommended by clinical guidelines, current evidence concerning its effectiveness mainly comes from some retrospective clinical trials with a relatively small cohort, most of which lack control arm. The evidence from randomized, placebo-controlled clinical trials is urgently needed. Furthermore, the optimal timing, dosage and administration route of TCZ need to be clarified. Apart from IL-6 signaling, other pro-inflammatory pathways may also contribute to the development of COVID-19. The efficacy of other agents such as siltuximab, anakinra and infliximab in the treatment of COVID-19 is worth further investigation, which may help to combat this pandemic eventually.

## Author Contributions

DL, TZ, and YW performed the literature search and manuscript drafting. LX supervised and revised the manuscript. All authors contributed to the article and approved the submitted version.

## Conflict of Interest

The authors declare that the research was conducted in the absence of any commercial or financial relationships that could be construed as a potential conflict of interest.

## References

[1] Tsatsakis A Petrakis D Nikolouzakis TK Docea AO Calina D Vinceti M. COVID-19, an opportunity to reevaluate the correlation between long-term effects of anthropogenic pollutants on viral epidemic/pandemic events and prevalence. Food Chem Toxicol. (2020) 141:111418. 10.1016/j.fct.2020.111418 32437891 PMC7211730

[2] Docea AO Tsatsakis A Albulescu D Cristea O Zlatian O Vinceti M A new threat from an old enemy: reemergence of coronavirus. Int J Mol Med. (2020) 45:1631–43. 10.3892/ijmm.2020.4555 32236624 PMC7169834

[3] Moore JB June CH. Cytokine release syndrome in severe COVID-19. Science. (2020) 368:473–4. 10.1126/science.abb8925 32303591

[4] Chen G Wu D Guo W Cao Y Huang D Wang H. Clinical and immunological features of severe and moderate coronavirus disease 2019. J Clin Invest. (2020) 130:2620–9. 10.1172/JCI137244 32217835 PMC7190990

[5] Jose RJ Manuel A. COVID-19 cytokine storm: the interplay between inflammation and coagulation. Lancet Respir Med. (2020) 8:e46–7. 10.1016/S2213-2600(20)30216-2 32353251 PMC7185942

[6] Aziz M Fatima R Assaly R. Elevated interleukin-6 and severe COVID-19: a meta-analysis. J Med Virol. (2020) 92:2283–5. 10.1002/jmv.25948 32343429 PMC7267383

[7] Gao Y Li T Han M Li X Wu D Xu Y. Diagnostic utility of clinical laboratory data determinations for patients with the severe COVID-19. J Med Virol. (2020) 92:791–6. 10.1002/jmv.25770 32181911 PMC7228247

[8] Chen R Sang L Jiang M Yang Z Jia N Fu W. Longitudinal hematologic and immunologic variations associated with the progression of COVID-19 patients in China. J Allergy Clin Immunol. (2020) 146:89–100. 10.1016/j.jaci.2020.05.003 32407836 PMC7212968

[9] Le RQ Li L Yuan W Shord SS Nie L Habtemariam BA FDA approval summary: tocilizumab for treatment of chimeric antigen receptor T cell-induced severe or life-threatening cytokine release syndrome. Oncologist. (2018) 23:943–7. 10.1634/theoncologist.2018-0028 29622697 PMC6156173

[10] Huang C Wang Y Li X Ren L Zhao J Hu Y. Clinical features of patients infected with 2019 novel coronavirus in Wuhan, China. Lancet. (2020) 395:497–506. 10.1016/S0140-6736(20)30183-5 31986264 PMC7159299

[11] Ruan Q Yang K Wang W Jiang L Song J Clinical predictors of mortality due to COVID-19 based on an analysis of data of 150 patients from Wuhan, China. Intensive Care Med. (2020) 46:846–8. 10.1007/s00134-020-05991-x 32125452 PMC7080116

[12] Zhou F Yu T Du R Fan G Liu Y Liu Z Clinical course and risk factors for mortality of adult inpatients with COVID-19 in Wuhan, China: a retrospective cohort study. Lancet. (2020) 395:1054–62. 10.1016/S0140-6736(20)30566-3 32171076 PMC7270627

[13] Xu Z Shi L Wang Y Zhang J Huang L Zhang C Pathological findings of COVID-19 associated with acute respiratory distress syndrome. Lancet Respir Med. (2020) 8:420–2. 10.1016/S2213-2600(20)30076-X 32085846 PMC7164771

[14] Shimizu M Clinical Features of Cytokine Storm Syndrome. In: Cron R Behrens E editors. Cytokine Storm Syndrome. Springer, Cham (2019). p. 31–41. 10.1007/978-3-030-22094-5_3

[15] Channappanavar R Perlman S. Pathogenic human coronavirus infections: causes and consequences of cytokine storm and immunopathology. Semin Immunopathol. (2017) 39:529–39. 10.1007/s00281-017-0629-x 28466096 PMC7079893

[16] Hunter CA Jones SA. IL-6 as a keystone cytokine in health and disease. Nat Immunol. (2015) 16:448–57. 10.1038/ni.3153 25898198

[17] Jones SA Jenkins BJ. Recent insights into targeting the IL-6 cytokine family in inflammatory diseases and cancer. Nat Rev Immunol. (2018) 18:773–89. 10.1038/s41577-018-0066-7 30254251

[18] Schmidt-Arras D Rose-John S. IL-6 pathway in the liver: from physiopathology to therapy. J Hepatol. (2016) 64:1403–15. 10.1016/j.jhep.2016.02.004 26867490

[19] Naugler WE Karin M. The wolf in sheep's clothing: the role of interleukin-6 in immunity, inflammation and cancer. Trends Mol Med. (2008) 14:109–19. 10.1016/j.molmed.2007.12.007 18261959

[20] Yamasaki K Taga T Hirata Y Yawata H Kawanishi Y Seed B. Cloning and expression of the human interleukin-6 (BSF-2/IFN beta 2) receptor. Science. (1988) 241:825–8. 10.1126/science.3136546 3136546

[21] Taga T Hibi M Hirata Y Yamasaki K Yasukawa K Matsuda T. Interleukin-6 triggers the association of its receptor with a possible signal transducer, gp130. Cell. (1989) 58:573–81. 10.1016/0092-8674(89)90438-8 2788034

[22] Hibi M Murakami M Saito M Hirano T Taga T Kishimoto T. Molecular cloning and expression of an IL-6 signal transducer, gp130. Cell. (1990) 63:1149–57. 10.1016/0092-8674(90)90411-7 2261637

[23] Kang S Tanaka T Narazaki M Kishimoto T. Targeting interleukin-6 signaling in clinic. Immunity. (2019) 50:1007–23. 10.1016/j.immuni.2019.03.026 30995492

[24] Schaper F Rose-John S. Interleukin-6: biology, signaling and strategies of blockade. Cytokine Growth Factor Rev. (2015) 26:475–87. 10.1016/j.cytogfr.2015.07.004 26189695

[25] Rose-John S. IL-6 trans-signaling via the soluble IL-6 receptor: importance for the pro-inflammatory activities of IL-6. Int J Biol Sci. (2012) 8:1237–47. 10.7150/ijbs.4989 23136552 PMC3491447

[26] Park A Iwasaki A. Type I and type III interferons - induction, signaling, evasion, and application to combat COVID-19. Cell Host Microbe. (2020) 27:870–8. 10.1016/j.chom.2020.05.008 32464097 PMC7255347

[27] Murakami M Kamimura D Hirano T. Pleiotropy and specificity: insights from the interleukin 6 family of cytokines. Immunity. (2019) 50:812–31. 10.1016/j.immuni.2019.03.027 30995501

[28] Blanco-Melo D Nilsson-Payant BE Liu WC Uhl S Hoagland D Moller R. Imbalanced host response to SARS-CoV-2 drives development of COVID-19. Cell. (2020) 181:1036–45. 10.1016/j.cell.2020.04.026 32416070 PMC7227586

[29] Xu B Fan CY Wang AL Zou YL Yu YH He C. Suppressed T cell-mediated immunity in patients with COVID-19: a clinical retrospective study in Wuhan, China. J Infect. (2020) 81:e51–60. 10.1016/j.jinf.2020.04.012 32315725 PMC7166040

[30] Wang F Hou H Luo Y Tang G Wu S Huang M. The laboratory tests and host immunity of COVID-19 patients with different severity of illness. JCI Insight. (2020) 5:e137799. 10.1172/jci.insight.137799 32324595 PMC7259533

[31] Diao B Wang C Tan Y Chen X Liu Y Ning L. Reduction and functional exhaustion of t cells in patients with coronavirus disease 2019 (COVID-19). Front Immunol. (2020) 11:827. 10.3389/fimmu.2020.00827 32425950 PMC7205903

[32] Chen Y Feng Z Diao B Rongshuai W Wang G Wang C The novel severe acute respiratory syndrome coronavirus 2 (SARS-CoV-2) directly decimates human spleens and lymph nodes. medRxiv. (2020). 10.1101/2020.03.27.20045427

[33] Yasukawa K Hirano T Watanabe Y Muratani K Matsuda T Nakai S. Structure and expression of human B cell stimulatory factor-2 (BSF-2/IL-6) gene. EMBO J. (1987) 6:2939–45. 10.1002/j.1460-2075.1987.tb02598.x 3500852 PMC553729

[34] Ma H Zeng W He H Zhao D Jiang D Zhou P. Serum IgA, IgM, and IgG responses in COVID-19. Cell Mol Immunol. (2020) 17:773–5. 10.1038/s41423-020-0474-z 32467617 PMC7331804

[35] Jones BE Maerz MD Buckner JH. IL-6: a cytokine at the crossroads of autoimmunity. Curr Opin Immunol. (2018) 55:9–14. 10.1016/j.coi.2018.09.002 30248523 PMC6286200

[36] Qin C Zhou L Hu Z Zhang S Yang S Tao Y. Dysregulation of immune response in patients with Coronavirus 2019 (COVID-19) in Wuhan, China. Clin Infect Dis. (2020) 71:762–8. 10.1093/cid/ciaa248 32161940 PMC7108125

[37] Li H Xiang X Ren H Xu L Zhao L Chen X. Serum amyloid A is a biomarker of severe Coronavirus disease and poor prognosis. J Infect. (2020) 80:646–55. 10.1016/j.jinf.2020.03.035 32277967 PMC7141628

[38] Qu D Liu J Lau CW Huang Y. IL-6 in diabetes and cardiovascular complications. Br J Pharmacol. (2014) 171:3595–603. 10.1111/bph.12713 24697653 PMC4128059

[39] Choy EH De Benedetti F Takeuchi T Hashizume M John MR Kishimoto T Translating IL-6 biology into effective treatments. Nat Rev Rheumatol. (2020) 16:335–45. 10.1038/s41584-020-0419-z 32327746 PMC7178926

[40] Newick K O'Brien S Moon E Albelda SM. CAR T cell therapy for solid tumors. Annu Rev Med. (2017) 68:139–52. 10.1146/annurev-med-062315-120245 27860544

[41] Alattar R Ibrahim T Shaar SH Abdalla S Shukri K Daghfal JN Tocilizumab for the treatment of severe coronavirus disease 2019. J Med Virol. (2020) 92:2042–9. 10.1002/jmv.25964 PMC7267594 32369191

[42] Luo P Liu Y Qiu L Liu X Liu D Li J. Tocilizumab treatment in COVID-19: a single center experience. J Med Virol. (2020) 92:814–8. 10.1002/jmv.25801 32253759 PMC7262125

[43] Xu X Han M Li T Sun W Wang D Fu B Effective treatment of severe COVID-19 patients with tocilizumab. Proc Natl Acad Sci USA. (2020) 117:10970–5. 10.1073/pnas.2005615117 32350134 PMC7245089

[44] Sciascia S Apra F Baffa A Baldovino S Boaro D Boero R Pilot prospective open, single-arm multicentre study on off-label use of tocilizumab in patients with severe COVID-19. Clin Exp Rheumatol. (2020) 38:529–32. 32359035

[45] Capra R De Rossi N Mattioli F Romanelli G Scarpazza C Sormani MP Impact of low dose tocilizumab on mortality rate in patients with COVID-19 related pneumonia. Eur J Intern Med. (2020) 76:31–5. 10.1016/j.ejim.2020.05.009 32405160 PMC7219361

[46] Toniati P Piva S Cattalini M Garrafa E Regola F Castelli F. Tocilizumab for the treatment of severe COVID-19 pneumonia with hyperinflammatory syndrome and acute respiratory failure: a single center study of 100 patients in Brescia, Italy. Autoimmun Rev. (2020) 19:102568. 10.1016/j.autrev.2020.102568 32376398 PMC7252115

[47] Colaneri M Bogliolo L Valsecchi P Sacchi P Zuccaro V Brandolino F. Tocilizumab for treatment of severe COVID-19 patients: preliminary results from SMAtteo Covid19 registry (SMACORE). Microorganisms. (2020) 8:695. 10.3390/microorganisms8050695 32397399 PMC7285503

[48] Roumier M Paule R Groh M Vallee A Ackermann F Interleukin-6 blockade for severe COVID-19. Ann Rheum Dis. (2020) 79:1277–85. 10.1101/2020.04.20.20061861 32620597 PMC7509526

[49] Klopfenstein T Zayet S Lohse A Balblanc JC Badie J Royer PY Tocilizumab therapy reduced intensive care unit admissions and/or mortality in COVID-19 patients. Med Mal Infect. (2020) 50:397–400. 10.1016/j.medmal.2020.05.001 32387320 PMC7202806

[50] Yang X Yu Y Xu J Shu H Xia J Liu H. Clinical course and outcomes of critically ill patients with SARS-CoV-2 pneumonia in Wuhan, China: a single-centered, retrospective, observational study. Lancet Respir Med. (2020) 8:475–81. 10.1016/S2213-2600(20)30079-5 32105632 PMC7102538

[51] Velazquez-Salinas L Verdugo-Rodriguez A Rodriguez LL Borca MV The role of interleukin 6 during viral infections. Front Microbiol. (2019) 10:1057 10.3389/fmicb.2019.01057 31134045 PMC6524401

[52] Yan L Zhang H Goncalves J Xiao Y Wang M Guo Y. An interpretable mortality prediction model for COVID-19 patients. Nat Mach Intel. (2020) 2:283–8. 10.1038/s42256-020-0180-733024092

[53] Zhang S Li L Shen A Chen Y Qi Z. Rational use of tocilizumab in the treatment of novel coronavirus pneumonia. Clin Drug Investig. (2020) 40:511–8. 10.1007/s40261-020-00917-3 32337664 PMC7183818

[54] Merad M Martin JC Pathological inflammation in patients with COVID-19: a key role for monocytes and macrophages. Nat Rev Immunol. (2020) 20:355–62. 10.1038/s41577-020-0331-4 32376901 PMC7201395

[55] Marfella R Paolisso P Sardu C Bergamaschi L D'Angelo EC Barbieri M. Negative impact of hyperglycaemia on tocilizumab therapy in Covid-19 patients. Diabetes Metab. (2020) 46:403–5. 10.1101/2020.04.29.20076570 32447102 PMC7241396

[56] Canziani LM Trovati S Brunetta E Testa A De Santis M Bombardieri E. Interleukin-6 receptor blocking with intravenous tocilizumab in COVID-19 severe acute respiratory distress syndrome: a retrospective case-control survival analysis of 128 patients. J Autoimmun. (2020) 114:102511. 10.1016/j.jaut.2020.102511 32713677 PMC7342030

